# Neuronal Sequence Models for Bayesian Online Inference

**DOI:** 10.3389/frai.2021.530937

**Published:** 2021-05-21

**Authors:** Sascha Frölich, Dimitrije Marković, Stefan J. Kiebel

**Affiliations:** Department of Psychology, Technische Universität Dresden, Dresden, Germany

**Keywords:** neuronal sequences, Bayesian inference, generative models, Bayesian brain hypothesis, predictive coding, hierarchy of time scales, recurrent neural networks, spatiotemporal trajectories

## Abstract

Various imaging and electrophysiological studies in a number of different species and brain regions have revealed that neuronal dynamics associated with diverse behavioral patterns and cognitive tasks take on a sequence-like structure, even when encoding stationary concepts. These neuronal sequences are characterized by robust and reproducible spatiotemporal activation patterns. This suggests that the role of neuronal sequences may be much more fundamental for brain function than is commonly believed. Furthermore, the idea that the brain is not simply a passive observer but an active predictor of its sensory input, is supported by an enormous amount of evidence in fields as diverse as human ethology and physiology, besides neuroscience. Hence, a central aspect of this review is to illustrate how neuronal sequences can be understood as critical for probabilistic predictive information processing, and what dynamical principles can be used as generators of neuronal sequences. Moreover, since different lines of evidence from neuroscience and computational modeling suggest that the brain is organized in a functional hierarchy of time scales, we will also review how models based on sequence-generating principles can be embedded in such a hierarchy, to form a generative model for recognition and prediction of sensory input. We shortly introduce the Bayesian brain hypothesis as a prominent mathematical description of how online, i.e., fast, recognition, and predictions may be computed by the brain. Finally, we briefly discuss some recent advances in machine learning, where spatiotemporally structured methods (akin to neuronal sequences) and hierarchical networks have independently been developed for a wide range of tasks. We conclude that the investigation of specific dynamical and structural principles of sequential brain activity not only helps us understand how the brain processes information and generates predictions, but also informs us about neuroscientific principles potentially useful for designing more efficient artificial neuronal networks for machine learning tasks.

## 1. Introduction

In the neurosciences, one important experimental and theoretical finding of recent years was that many brain functions can be described as predictive (Rao and Ballard, 1999; Pastalkova et al., 2008; Friston and Kiebel, 2009; Aitchison and Lengyel, 2017). This means that the brain not only represents current states of the environment but also potential states of the future to adaptively select its actions and behavior. For such predictions, one important feature of neuronal dynamics is their often-observed sequence-like structure. In this review, we will present evidence that sequence-like structure in neuronal dynamics is found over a wide range of different experiments and different species. In addition, we will also review models for such sequence-like neuronal dynamics, which can be used as generative models for Bayesian inference to compute predictions. To familiarize readers of different backgrounds with each of these topics, we first briefly give an overview of the topics of sequences, predictions, hierarchical structure, the so-called Bayesian brain hypothesis and provide a more precise definition of the kind of sequence-like neuronal dynamics that we consider in this review.

### 1.1. Sequences in the Brain

The brain is constantly receiving spatiotemporally structured sensory input. This is most evident in the auditory domain where, when listening to human speech, the brain receives highly structured, sequential input in the form of phonemes, words, and sentences (Giraud and Poeppel, 2012). Furthermore, even in situations which apparently provide only static sensory input, the brain relies on spatiotemporally structured coding. For example, when observing a static visual scene, the eyes constantly perform high-frequency micro-oscillations and exploratory saccades (Martinez-Conde et al., 2004; Martinez-Conde, 2006), which renders the visual input spatiotemporally structured, and yet the visual percepts appear stationary. Another example is olfaction, where in animal experiments, it has been shown that neurons in the olfactory system respond to a stationary odor with an elaborate temporal coding scheme (Bazhenov et al., 2001; Jones et al., 2007). In the state space of those neurons, their activity followed a robust and reproducible trajectory, a neuronal sequence (see Table 1), which was specific to the presented odor. Similarly, in a behavioral experiment with monkeys, spatial information of an object was encoded by a dynamical neural code, although the encoded relative location of the object remained unchanged (Crowe et al., 2010). In other words, there is evidence that the brain recognizes both dynamic and static entities in our environment on the basis of sequence-like encoding.

**Table 1:** Glossary.

Neuronal sequence	Spatiotemporal patterns of neuronal activity that encode stimulus properties, abstract concepts, or motion signals (see Figure 1). Can be described by a specific, sequential trajectory in the so-called state space of the system, see also Figure 3 for an example.
State space/Phase space	A multidimensional space that encompasses all possible states a system can be in. Every possible state is defined by a unique point in the space.
Continuodiscrete dynamics/Trajectory	Reproducible spatiotemporal trajectories characterized by discrete points in state space (see Figure 3).
Winnerless Competition (WLC)	Type of dynamic behavior of a system where the system shortly settles into a stable or metastable state before being forced away from it (by internal or external mechanisms) (see Figures 3, 6).
Metastable state/Saddle state	A state in the state space of a dynamical system. A metastable state of a system is stable in some directions and unstable in others. A saddle point is a metastable point where the first derivative vanishes.
Stable heteroclinic channel (SHC)	Type of dynamic behavior of a system where the system goes through a succession of saddle points (metastable states) forming heteroclinic state-space trajectories (orbits). Importantly, small deviations from those trajectories will not diverge away from the heteroclinic orbit. See section 2.2.2.
Heteroclinic orbit/Trajectory	A path in the state space of a system that connects two equilibrium points.
Limit cycle	Attractor type occurring in some complex dynamical systems. Closed, continuous trajectory in state space with fixed period and amplitude. The regular firing behavior of neurons can be described by limit cycle behavior. See section 2.2.1.
Synfire chain	A feed-forward neuronal network architecture. See section 2.1.

**Figure 1:**
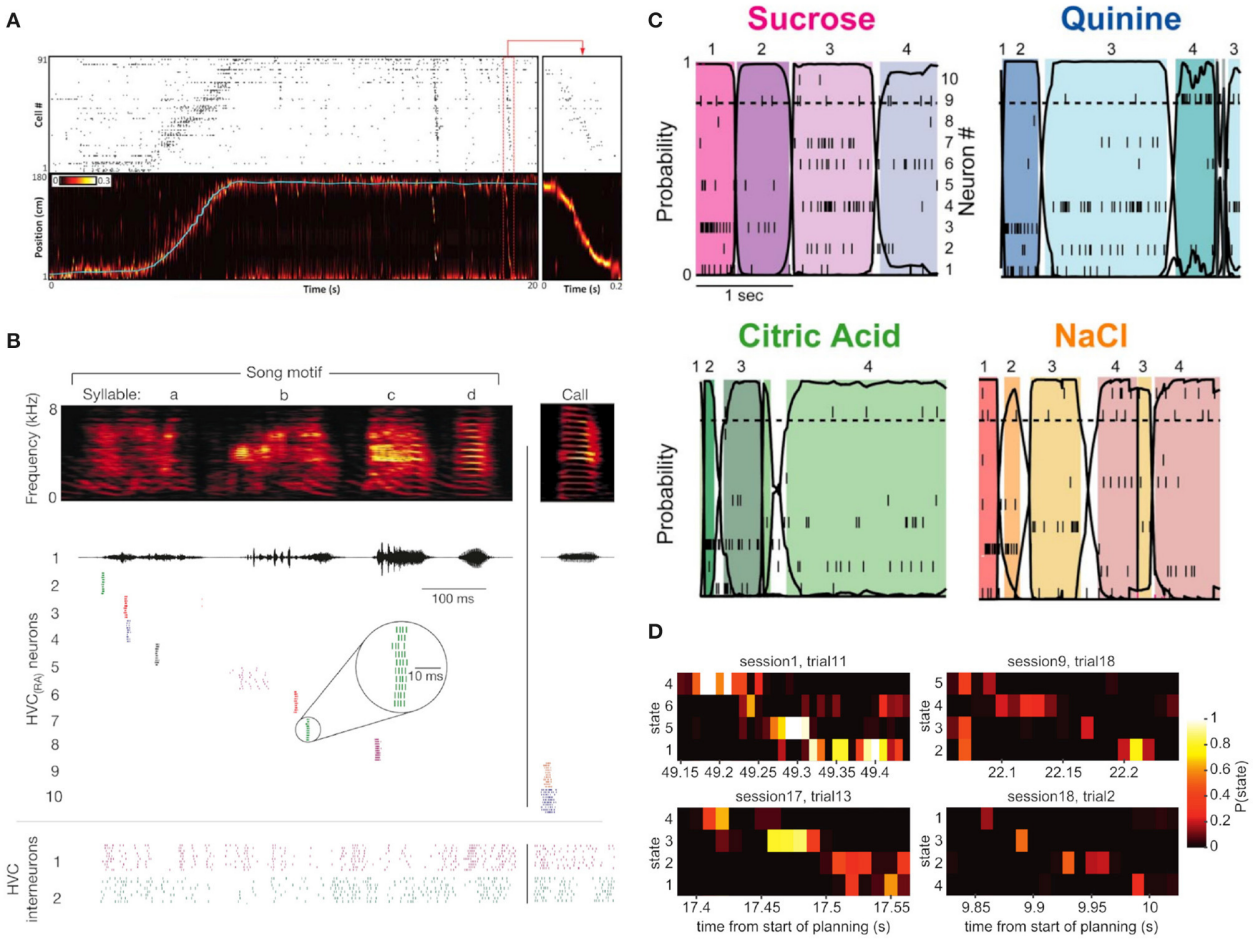
Four illustrative examples of sequential neuronal activity in different paradigms and experimental contexts. **(A)** Sequential activation of rat hippocampal cells are found during action and in rest phases after the behavioral tasks. The top plot shows the spiking histogram of 91 hippocampal cells during a rat's trip along a physical track. The bottom panel shows the rat's actual position on the track (blue line) against the position inferred from the spiking pattern of its hippocampal cells. After the traversal of the track, hippocampal cells “replayed” their activation sequence in reverse during a short ripple event (red box, enlarged in the box on the right). Figure adapted from Pfeiffer (2020) (Copyright 1999–2019 John Wiley & Sons, Inc.). **(B)** Zebra finches are songbirds whose songs consist of highly consistent so-called song motifs. Here, the activations of ten different HVC_(RA)_ neurons and two HVC interneurons in the HVC nucleus of the zebra finch brain during ten renditions of the same song motif are shown. HVC_(RA)_ project from the HVC nucleus to the RA nucleus in the birdbrain, and exhibit precise and reproducible firing sequences during the rendition of a song. Adapted from Hahnloser et al. Hahnloser et al. (2002) with permission from Springer Nature. **(C)** Firing patterns of neurons in the gustatory cortex of rats *in vivo* when presented with four different odors. The sequential switching of states of a hidden Markov model (HMM, see section 3.1) was characteristic of the presented aroma. For each of the four odors, the different color hues represent different HMM states that were inferred based on the data. Adapted from Jones et al. (2007) (Copyright 2007 National Academy of Sciences, U.S.A.). **(D)** Evidence for fast sequence representation in human participants during planning of a trajectory through task state space, see Kurth-Nelson et al. (2016) for details. The four examples, each for a different participant, show evidence of brain activity, as measured with magnetoencephalography (MEG), to quickly transition through task state space with roughly 40 ms duration for each sequence element. Figure taken from Kurth-Nelson et al. (2016).

Neuronal sequences have been reported in a wide range of experimental contexts. For example, in the hippocampus of mice and rats (MacDonald et al., 2011; Pastalkova et al., 2008; Bhalla, 2019; Skaggs and McNaughton, 1996; Dragoi and Tonegawa, 2011), the visual cortex of cats and rats (Kenet et al., 2003; Ji and Wilson, 2007), the somatosensory cortex of mice (Laboy-Juárez et al., 2019), the parietal cortex of monkeys and mice (Crowe et al., 2010; Harvey et al., 2012), the frontal cortex of monkeys (Seidemann et al., 1996; Abeles et al., 1995; Baeg et al., 2003), the gustatory cortex of rats (Jones et al., 2007), the locust antennal lobe (Bazhenov et al., 2001), specific song-related areas in the brain of songbirds (Hahnloser et al., 2002), and the amygdala of monkeys (Reitich-Stolero and Paz, 2019), among others. Even at the cellular level, there is evidence of sequence-processing capacities of single neurons (Branco et al., 2010). Neuronal sequences seem to serve a variety of different purposes. While sequences in specific brain regions drive the spatiotemporal motor patterns during behavior like birdsong rendition (Hahnloser et al., 2002) (Figure 1B), in other studies of different brain areas and different species, neuronal sequences were found to encode stationary stimuli (Seidemann et al., 1996; Bazhenov et al., 2001) and spatial information (Crowe et al., 2010), to represent past experience (Skaggs and McNaughton, 1996) (see also Figure 1A), and to be involved with both working memory and memory consolidation (MacDonald et al., 2011; Harvey et al., 2012; Skaggs and McNaughton, 1996). Behaviorally relevant neuronal sequences were reported to occur before the first execution of a task (Dragoi and Tonegawa, 2011), and in some behavioral tasks sequences were found to be predictive of future behavior (Abeles et al., 1995; Pastalkova et al., 2008).

As these findings show, neuronal sequences can be measured in different species, in different brain areas and at different levels of observation, where the expression of these sequences depends on the measurement and analysis method. A neuronal sequence can appear as the successive spiking of neurons (Figures 1A,B), or the succession of more abstract compound states (Figure 1C), or in yet different forms, depending on the experimental approach. For example, evidence for sequences can also be found with non-invasive cognitive neuroscience methods like magnetoencephalography (MEG) as shown in Figure 1D. Given these very different appearances of experimentally observed neuronal sequences, it is clear that an answer to the question of “What is a neuronal sequence?” depends on the experimental setup. In the context of this article, we understand a “neuronal sequence” quite broadly as any kind of robust and reproducible spatiotemporal trajectory, where stimulus properties, abstract concepts, or motion signals are described by a specific trajectory in the state space of the system (see Table 1). The brain may use such trajectory representations, whose experimental expressions are measured as neuronal sequences, to form a basis for encoding the spatiotemporal structure of sensory stimuli (Buonomano and Maass, 2009) and the statistical dependencies between past, present, and future (Friston and Buzsáki, 2016). Here, we will review evidence for this type of encoding and discuss some of the implications for our understanding of the brain's capacity to perform probabilistic inference, i.e., recognition based on spatiotemporally structured sensory input.

### 1.2. Hierarchies in the Brain

The brain's structure and function are often described with reference to a hierarchical organization, which we will cover in more detail in section 3.2. Human behavior can be described as a hierarchically structured process (Lashley and Jeffress, 1951; Rosenbaum et al., 2007; Dezfouli et al., 2014), as can memory, where the grouping of information-carrying elements into chunks constitutes a hierarchical scheme (Bousfield, 1953; Miller, 1956; Fonollosa et al., 2015). Similarly, the perception and recognition of spatiotemporally structured input can be regarded as a hierarchical process. For example, percepts, such as the observation of a walking person can be regarded as percepts of higher order (“walking person”), as they emerge from the combination of simpler, lower order percepts, e.g., a specific sequence of limb movements. Critically, the concept “someone walking” is represented at a slower time scale as compared to the faster movements of individual limbs that constitute the walking. There is emerging evidence that the brain is structured and organized hierarchically along the relevant time scales of neuronal sequences (e.g., Murray et al., 2014; Hasson et al., 2008; Cocchi et al., 2016; Mattar et al., 2016; Gauthier et al., 2012; Kiebel et al., 2008). Such a hierarchy allows the brain to model the causal structure of its sensory input and form predictions at slower time scales (“someone walking”) by representing trajectories capturing the dynamics of its expected spatiotemporal sensory input at different time scales, and by representing causal dependencies between time scales. This allows for inference about the causes of sensory input in the environment, as well as for inference of the brain's own control signals (e.g., motor actions). In this paper, we will review some of the experimental evidence and potential computational models for sequence generation and inference.

In the following section 1.3 we will first give a short introduction to the Bayesian brain hypothesis and the basic concept of the brain as a predictor of its environment. In section 1.4 we will go into more detail about the question “What is a sequence?” and will further discuss the trajectory representation. In section 2, we will provide an overview of several dynamical principles that might underlie the generation of neuronal trajectories in biological networks. Importantly, we are going to focus on general dynamical network principles that may underlie sequence generation, and which may differentiate types of sequence-generating networks. We are therefore not going to cover the vast field of sequence learning (e.g., Sussillo and Abbott, 2009; Tully et al., 2016; Lipton et al., 2015; Wörgötter and Porr, 2005), which mainly investigates neurobiologically plausible learning rules and algorithms that can lead to neuronal sequences, and thus possibly to the network types discussed in this article. In section 3, we review some approaches in which sequences are used to model recognition of sensory input. To highlight the relevance of sequence generators to a large variety of problems, we will visit methods and advances in computer science and machine learning, where structured artificial recurrent neural networks (RNNs) that are able to generate spatiotemporal activity patterns are used to perform a range of different computational tasks. This will however only serve as a rough and incomplete overview over some common machine learning methods, and we will not cover methods like Markov Decision Processes (Feinberg and Shwartz, 2012) and related approaches, as an overview of research on sequential decision making is beyond the scope of this review. Finally, we will briefly discuss functional hierarchies in the brain and in machine learning applications. A glossary of technical terms that we will use in the review can be found in Table 1.

### 1.3. The Bayesian Brain Hypothesis

**Figure 2:**
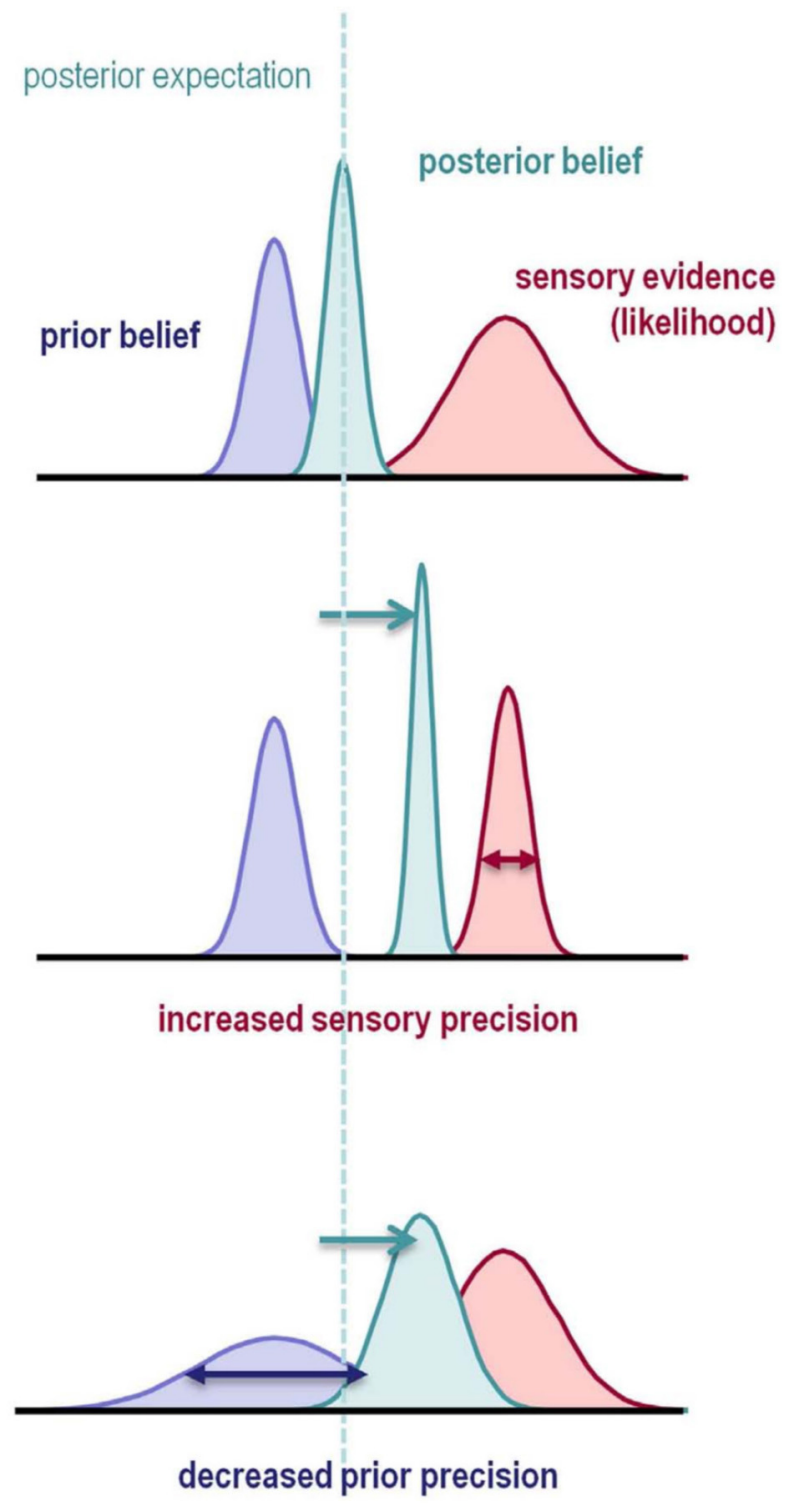
Illustration of Bayesian Inference. The prior belief (blue) about a state is updated by sensory evidence (red) represented by the likelihood function. The updated belief is the posterior belief (turquoise), which will serve as the prior belief in the next updating step. Each row illustrates how the shape of the prior distribution and the likelihood influence the inference process. Both an increase in likelihood precision (inverse variance), and a decrease in prior precision result in a posterior belief which is more biased toward the sensory evidence. This is illustrated by a deviation of the posterior toward the sensory evidence and away from the prior belief (dashed line and arrows). In the Bayesian predictive coding framework (Friston and Kiebel, 2009; Rao and Ballard, 1999), inference naturally minimizes the prediction error, defined as the difference between expected and observed outcomes. Figure reprinted from Adams et al. (2013).

Dating back to Hermann von Helmholtz in the 19th century, the idea that the brain performs statistical inference on its sensory input to infer the underlying probable causes of that same input (Helmholtz, 1867), started gaining considerable traction toward the end of the 20th century and had a strong influence on both computer science and neuroscience (Hinton and Sejnowski, 1983; Dayan et al., 1995; Wolpert et al., 1995; Friston, 2005; Friston et al., 2006; Beck et al., 2008; see also Rao and Ballard, 1999; Ernst and Banks, 2002; Körding and Wolpert, 2004). In particular, research into this interpretation of brain function led to the formulation of the Bayesian brain hypothesis (Knill and Pouget, 2004; Doya et al., 2007; Friston, 2010). The Bayesian brain hypothesis posits that aspects of brain function can be described as equivalent to Bayesian inference based on a causal generative model of the world, which models the statistical and causal regularities of the environment. In this framework, recognition is modeled as Bayesian inversion of the generative model, which assigns probabilities, that is, beliefs to different states of the world based on perceived sensory information. This process of Bayesian inference is hypothesized to be an appropriate basis for the mathematical description of most, if not all, brain functions (Friston, 2010; Knill and Pouget, 2004). Although the hypothesis that the brain is governed by Bayesian principles has met with criticism since human behavior does not always appear to be Bayes-optimal (Rahnev and Denison, 2018; Soltani et al., 2016), and because the definition of Bayes-optimality can be ambiguous (Colombo and Seriès, 2012), there is growing evidence that human behavior can indeed be explained by Bayesian principles (Figure 2) (Ernst and Banks, 2002; Körding and Wolpert, 2004; Weiss et al., 2002; Feldman, 2001), and that even phenomena like mental disorders might be explained by Bayesian mechanisms (Adams et al., 2013; Leptourgos et al., 2017; Fletcher and Frith, 2009) (see Knill and Pouget, 2004 and Clark, 2013 for reviews on the Bayesian brain hypothesis). How Bayesian inference is achieved in the human brain is an ongoing debate, and it has been proposed that the corresponding probabilities are encoded on a population level (Zemel et al., 1998; Beck et al., 2008) or on single-neuron level (Deneve, 2008).

Under the Bayesian view, model inversion, i.e., recognition, satisfies Bayes' theorem, which states that the optimal posterior belief about a state is proportional to the generative model's prior expectation about the state multiplied by the probability of the sensory evidence under the generative model. In Bayesian inference, prior expectation, posterior belief, and sensory evidence are represented as probability distributions and accordingly called *prior distribution, posterior distribution*, and *likelihood* (Figure 2). The posterior can be regarded as an updated version of the prior distribution, and will act as the prior in the next inference step. Importantly, the prior is part of the generative model as different priors could lead to qualitatively different expectations (Gelman et al., 2017).

The quality of the inference, that is, the quality of the belief about the hidden states of the world, is dependent on the quality of the agent's generative model, and the appropriateness of a tractable (approximate) inference scheme. In this review paper, we suggest that good generative models of our typical environment should generate, that is, expect sequences, and that such a sequence-like representation of environmental dynamics is used to robustly perform tractable inference on spatiotemporally structured sensory data.

The theory of predictive coding suggests that the equivalent of an inversion of the generative model in the cortex is achieved in a hierarchical manner by error-detecting neurons which encode the difference between top-down predictions and sensory input (Friston and Kiebel, 2009; Rao and Ballard, 1999; Aitchison and Lengyel, 2017) (Figure 2). The fact that sequences in specific contexts appear to have predictive properties (Abeles et al., 1995; Pastalkova et al., 2008) is interesting in light of possible combinations of the frameworks of predictive coding and the Bayesian brain hypothesis (Knill and Pouget, 2004; Doya et al., 2007; Friston, 2010). One intriguing idea is that the brain's internal representations and predictions rely on sequences of neuronal activity (FitzGerald et al., 2017; Kiebel et al., 2009; Hawkins et al., 2009). Importantly, empirical evidence suggests that these approximate representations are structured in temporal and functional hierarchies (see sections 1.2 and 3.2) (Koechlin et al., 2003; Giese and Poggio, 2003; Botvinick, 2007; Badre, 2008; Fuster, 2004). Combining the Bayesian brain hypothesis with the hierarchical aspect of predictive coding provides a theoretical basis for computational mechanisms that drive a lifelong learning of the causal model of the world (Friston et al., 2014). Examples for how these different frameworks can be combined can be found in Yildiz and Kiebel (2011) and Yildiz et al. (2013).

As an example of a tight connection between prediction and sequences, one study investigating the electrophysiological responses in the song nucleus HVC of bengalese finch (Bouchard and Brainard, 2016) found evidence for an internal prediction of upcoming song syllables, based on sequential neuronal activity in HVC. As another example, a different study investigating single-cell recordings of neurons in the rat hippocampus found that sequences of neuronal activations during wheel-running between maze runs were predictive of the future behavior of the rats, including errors (Pastalkova et al., 2008). This finding falls in line with other studies showing that hippocampal sequences can correlate with future behavior (Pfeiffer, 2020).

### 1.4. What Are Sequences?

What does it mean to refer to neuronal activity as sequential? In the most common sense of the word, a sequence is usually understood as the serial succession of discrete elements or states. Likewise, when thinking of sequences, most people intuitively think of examples like “A, B, C,…” or “1, 2, 3,….” However, when extending this discrete concept to neuronal sequences, there are only few compelling examples where spike activity is readily interpretable as a discrete sequence, like the “domino-chain” activation observed in the birdbrain nucleus HVC (Hahnloser et al., 2002) (Figure 1B). As mentioned before, we will use the word “sequence” to describe robust and reproducible spatiotemporal trajectories, which encode information to be processed or represented. Apart from the overwhelming body of literature reporting sequences in many different experimental settings (section 1.1), particularly interesting are the hippocampus (Bhalla, 2019; Pfeiffer, 2020) and entorhinal cortex (Zutshi et al., 2017; O'Neill et al., 2017). Due to the strong involvement of the hippocampus and the entorhinal cortex with sequences, the idea that neuronal sequences are also used in brain areas directly connected to them is not too far-fetched. For example, hippocampal-cortical interactions are characterized by sharp wave ripples (Buzsáki, 2015), which are effectively compressed spike sequences. Recent findings suggest that other cortical areas connected to the hippocampus use grid-cell like representations similar to space representation in the entorhinal cortex (Constantinescu et al., 2016; Stachenfeld et al., 2017). This is noteworthy because grid cells have been linked to sequence-like information processing (Zutshi et al., 2017; O'Neill et al., 2017). This suggests that at least areas connected to the hippocampus and entorhinal cortex are able to decode neuronal sequences.

**Figure 3:**
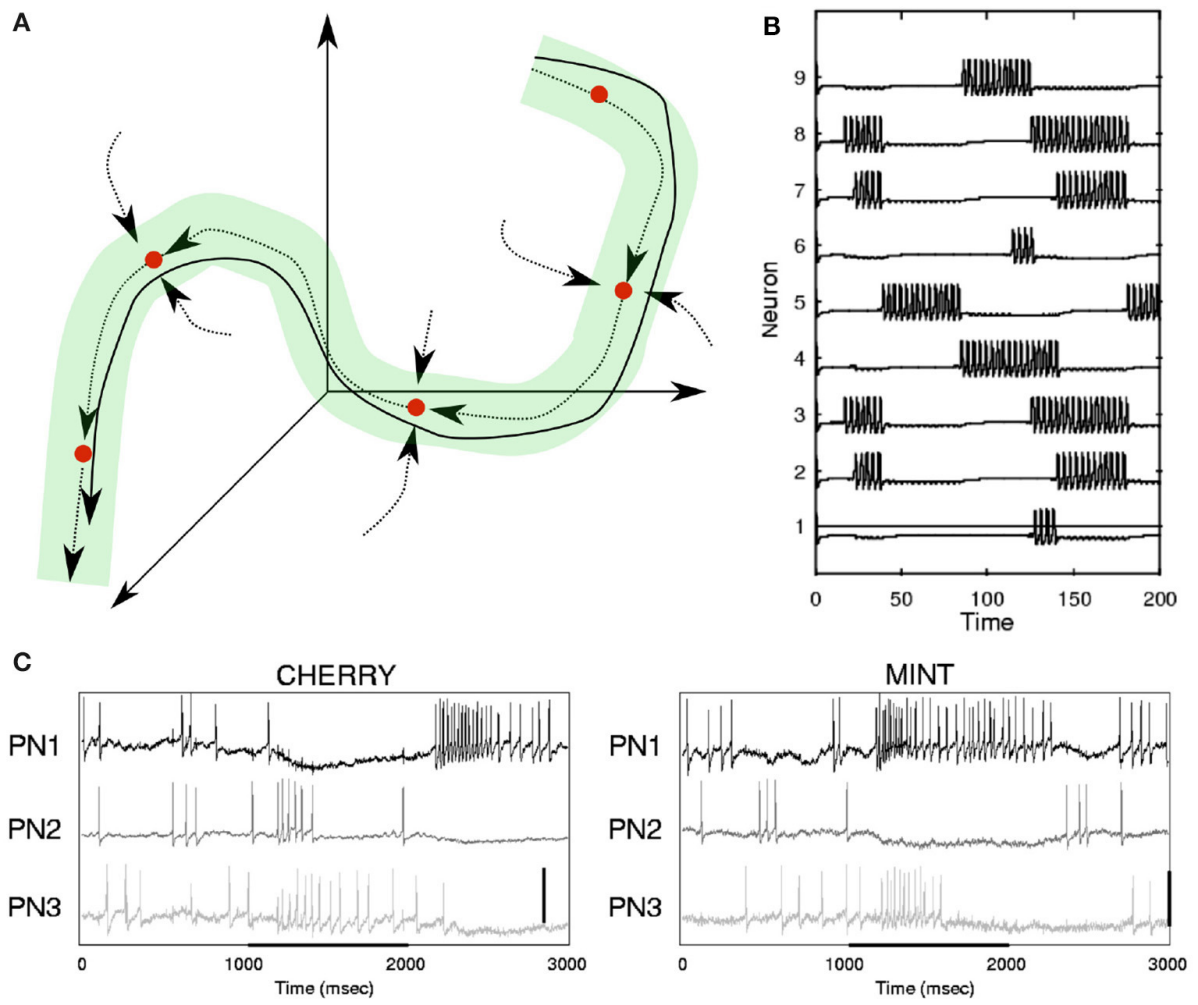
**(A)** Illustration of continuodiscrete dynamics based on Stable Heteroclinic Channels (SHC, see section 2.2.2 and Table 1). The solid line represents a continuous heteroclinic trajectory in three-dimensional phase space and the dotted lines indicate invariant manifolds between saddle states (see Table 1). The green tube illustrates a Stable Heteroclinic Channel. All heteroclinic trajectories originating in the SHC will remain inside of it. This is a type of WLC dynamics. **(B)** Simulation of an SHC-trajectory based on Lotka-Volterra dynamics, where a point in phase space determines the firing rate of each neuron. **(C)** Neuronal responses to odor representation in the locust brain. **(B,C)** Are adapted from Rabinovich et al. (2001). Copyright (2001) by the American Physical Society.

The example of odor recognition shows that sequences are present even in circumstances where one intuitively would not expect them (Figure 1C). This very example does also show an interesting gap between a continuous and a discrete type of representation: The spatiotemporal trajectory is of a continuous nature, while the representation of the odor identity is characterized by discrete states and at a slower time scale. This gap also presents itself on another level. While we understand the term “neuronal sequence” to refer to a robust and reproducible spatiotemporal trajectory, in many cases these continuous state-space trajectories appear as a succession of quasi-discrete states (Abeles et al., 1995; Seidemann et al., 1996; Mazor and Laurent, 2005; Jones et al., 2007). In order to emphasize this interplay between continuous dynamics and discrete points we will denote such dynamics as *continuodiscrete* (see Table 1). In continuodiscrete dynamics, robust, and reproducible spatiotemporal trajectories are characterized by discrete points in state-space. As an example, in Figure 1C one can see the response of *in vivo* neurons in the gustatory cortex of rats, which is determined by the odor that is presented to the animal. The activity patterns of the neurons were analyzed with a hidden Markov model which revealed that the activity of the neuron ensemble can be described as a robust succession of discrete Markov states, where the system remains in a state for hundreds of milliseconds before quickly switching to another discrete state. These sequential visits to discrete states and the continuous expression of these states, specifically the switching between them, in terms of fast neuronal dynamics (here spiking neurons) is what we consider as continuodiscrete dynamics. Similar observations have been made in other experiments (Abeles et al., 1995; Seidemann et al., 1996; Mazor and Laurent, 2005; Rabinovich et al., 2001; Rivera et al., 2015) (see also Figure 3). The discrete states of a continuodiscrete sequence can be for example stable fixed points (Gros, 2009), or saddle points (Rabinovich et al., 2006, 2001) of the system, or simply points along a limit cycle trajectory (Yildiz and Kiebel, 2011; Yildiz et al., 2013), depending on the modeling approach (see section 2). Depending on the dynamical model, the system might leave a fixed point due to autonomously induced destabilization (Gros, 2007, 2009), noise (Rabinovich et al., 2006, 2001), or external input (Kurikawa and Kaneko, 2015; Toutounji and Pipa, 2014; Rivera et al., 2015; Hopfield, 1982).

Concepts similar to continuodiscrete trajectories have been introduced before. For example, in winner-less competition (WLC) (Rabinovich et al., 2000; Afraimovich et al., 2004b; Rabinovich et al., 2008), a system moves from one discrete metastable fixed-point (see Table 1) of the state space to the next, never settling for any state, similar to the fluctuations in a Lotka-Volterra system (Rabinovich et al., 2001) (see Figure 3). In winner-take-all (WTA) dynamics, like during memory recall in a Hopfield network (Hopfield, 1982), the system is attracted to one fixed point in which it will settle. Both WLC and WTA are thus examples of continuodiscrete dynamics. The concept of continuodiscrete dynamics also allows for dynamics which are characterized by an initial alteration between discrete states, before settling into a final state, as for example in Rivera et al. (2015). In section 2, we will look at different ways to model continuodiscrete neuronal dynamics.

For the brain, representing continuodiscrete trajectories seems to combine the best of two worlds: Firstly, the representation of discrete points forms the basis for the generalization and categorization of the sequence. For example, for the categorization of a specific movement sequence, it is not necessary to consider all the details of the sensory input, as it is sufficient to categorize the sequence type (dancing, walking, running) by recognizing the sequence of discrete points, as e.g., in Giese and Poggio (2003). Secondly, the brain requires a way of representing continuous dynamics to not miss important details. This is because key information can only be inferred by subtle variations within a sequence, as is often the case in our environment. For instance, when someone is talking, most of the speech content, i.e., what is being said, is represented by discrete points that describe a sequence of specific vocal tract postures. Additionally, there are subtle variations in the exact expression of these discrete points and the continuous dynamics connecting them, which let us infer about otherwise hidden states like the emotional state of the speaker (Birkholz et al., 2010; Kotz et al., 2003; Schmidt et al., 2006). Some of these subtle variations in the sensory input may be of importance to the brain, while others are not. For example, when listening to someone speaking, slight variations in the speaker's talking speed or pitch of voice might give hints about her mood, state of health, or hidden intentions. In other words, representing sensory input as continuodiscrete trajectories enables the recognition of invariances of the underlying movements without losing details.

There is growing evidence that sequences with discrete states like fixed points are a fundamental feature of cognitive and perceptual representations (e.g., Abeles et al., 1995; Seidemann et al., 1996; Mazor and Laurent, 2005; Jones et al., 2007). This feature may be at the heart of several findings in the cognitive sciences which suggest that human perception is chunked into discrete states, see VanRullen and Koch (2003) for some insightful examples. Assuming that the brain uses some form of continuodiscrete dynamics to model sensory input, we will next consider neuronal sequence-generating mechanisms that may implement such dynamics and act as a generative model for recognition of sensory input. Importantly, as we are interested in generative models of sequential sensory input, we will only consider models that have the ability to autonomously generate sequential activity. Therefore, we are not going to discuss models where sequential activity is driven by sequential external input, as in models of non-autonomous neural networks (Toutounji and Pipa, 2014), or in models where intrinsic sequential neural activity is disrupted by bifurcation-inducing external input (Kurikawa and Kaneko, 2015).

## 2. Neuronal Network Models as Sequence Generators

In order to explain sequential neuronal activity in networks of biological neurons, several models have been proposed, some of which we are going to review in the following sections. As this paper aims at a general overview of neuronal sequence-generating mechanisms and less at a detailed analysis, we will not cover the details and nuances of the presented dynamical models and refer the interested reader to the references given in the text.

### 2.1. Synfire Chains

**Figure 4:**
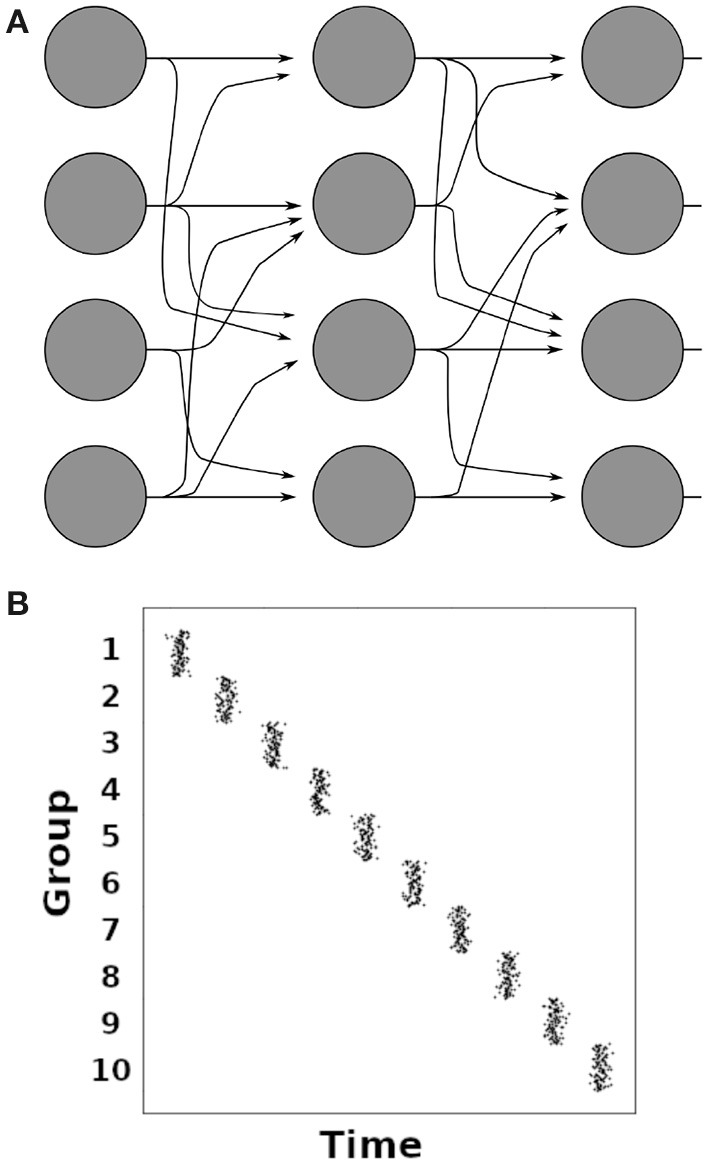
**(A)** Illustration of a synfire chain between groups of neurons (filled circles). Arrows indicate excitatory connections. **(B)** Illustration of a spiking histogram of neurons in a synfire chain with 10 groups of 100 neurons each. The average time interval between the firing of two adjacent groups corresponds to one synaptic delay.

Synfire chains are concatenated groups of excitatory neurons with convergent-divergent feed-forward connectivity, as illustrated in Figure 4A (Abeles, 1991; Diesmann et al., 1999). Synchronous activation of one group leads to the activation of the subsequent group in the chain after one synaptic delay (Figure 4B). It has been shown that the only stable operating mode in synfire chains is the synchronous mode where all neurons of a group spike in synchrony (Litvak et al., 2003). Synfire chains create sequences that are temporally highly precise (Abeles, 1991; Diesmann et al., 1999). Such temporally precise sequences have been observed in slices of the mouse primary visual cortex and in V1 of anaesthetized cats (Ikegaya et al., 2004), as well as in the HVC nucleus of the bird brain during song production (Hahnloser et al., 2002; Long et al., 2010), and in the frontal cortex of behaving monkeys (Prut et al., 1998; Abeles and Gat, 2001). While synfire chains make predictions that agree well with these observations, a striking mismatch between synfire chains and neuronal networks in the brain is the absence of recurrent connections in the synfire chain's feed-forward architecture. Modeling studies have shown that sequential activation similar to synfire chain activity can be achieved by changing a small fraction of the connections in a random neural network (Rajan et al., 2016; Chenkov et al., 2017), and that synfire chains can emerge in self-organizing recurrent neural networks under the influence of multiple interacting plasticity mechanisms (Zheng and Triesch, 2014). Such fractional changes of network connections were used to implement working memory (Rajan et al., 2016) or give a possible explanation for the occurrence of memory replay after one-shot learning (Chenkov et al., 2017). Such internally generated sequences have been proposed as a mechanism for memory consolidation, among other things (see Pezzulo et al., 2014 for a review).

### 2.2. Attractor Networks

#### 2.2.1. Limit Cycles

**Figure 5:**
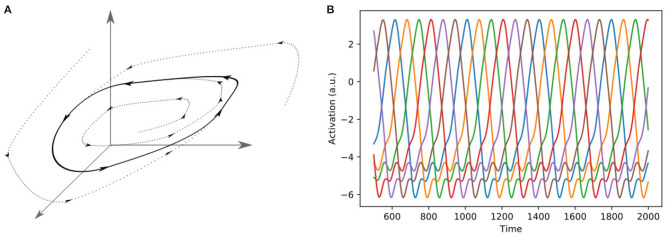
Two different representations of a limit cycle. **(A)** A Limit cycle in three-dimensional phase space. In the case of a neuronal network, the dimensions of the phase space can be interpreted as the firing rates of the neurons. **(B)** Representation of a six-dimensional limit cycle as alternating activations of six different neurons.

Limit cycles are stable attractors in the phase space of a system, and they occur in practically every physical domain (Strogatz, 2018). A limit cycle is a closed trajectory, with fixed period and amplitude (Figure 5). Limit cycles occur frequently in biological and other dynamical systems, and the beating of the heart, or the periodic firing of a pacemaker neuron are examples of limit cycle behavior (Strogatz, 2018). They are of great interest to theoretical neuroscience, as periodic spiking activity can be represented by limit cycles, both on single-cell level (Izhikevich, 2007) and population level (Berry and Quoy, 2006; Jouffroy, 2007; Mi et al., 2017). They also play an important role in the emulation of human motion in robotics. While there are numerous ways to model human motion, one interesting approach is that of *dynamic motion primitives* (DMPs) (Schaal et al., 2007), which elegantly unifies the two different kinds of human motion, rhythmic and non-rhythmic motion, in one framework. The main idea of DMPs is that the limbs move as if they were pulled toward an attractor state. In the case of rhythmic motion, the attractor is given by a limit cycle, while in the case of motion strokes the attractor is a discrete point in space (Schaal et al., 2007). In Kiebel et al. (2009), Yildiz and Kiebel (2011), and Yildiz et al. (2013), the authors used a hierarchical generative model of sequence-generators based on limit cycles to model the generation and perception of birdsong and human speech.

#### 2.2.2. Heteroclinic Trajectories

Another approach to modeling continuodiscrete dynamics are heteroclinic networks (Ashwin and Timme, 2005; Rabinovich et al., 2008) (see also Table 1). A heteroclinic network is a dynamical system with semi-stable states (saddle points) which are connected by invariant manifolds, so-called heteroclinic connections. Networks of coupled oscillators have been shown to give rise to phenomena like heteroclinic cycles (Ashwin and Swift, 1992; Ashwin et al., 2007). It has therefore been proposed that neuronal networks exhibit such heteroclinic behavior as well, which has been verified using simulations of networks of globally coupled Hodgkin-Huxley neurons (Hansel et al., 1993a,b; Ashwin and Borresen, 2004). Interestingly, heteroclinic networks can be harnessed to perform computational tasks (Ashwin and Borresen, 2005; Neves and Timme, 2012), and it has been shown that it is possible to implement any logic operation within such a network (Neves and Timme, 2012). Furthermore, the itinerancy in a heteroclinic network can be guided by external input, where the trajectory of fixed points discriminates between different inputs (Ashwin et al., 2007; Neves and Timme, 2012), which means that different inputs are encoded by different trajectories in phase space.

While theoretical neuroscience has progressed with research on heteroclinic behavior of coupled neural systems, concrete biological evidence is still sparse, as this requires a concrete and often complex mathematical model which is often beyond the more directly accessible research questions in biological science. Despite this, heteroclinic behavior has been shown to reproduce findings from single-cell recordings in insect olfaction (Rabinovich et al., 2001; Rivera et al., 2015) and olfactory bulb electroencephalography (EEG) in rabbits (Breakspear, 2001). Another study replicated the chaotic hunting behavior of a marine mollusk based on an anatomically plausible neuronal model with heteroclinic winnerless competition (WLC) dynamics (Varona et al., 2002), which is closely related to the dynamic alteration between states in a heteroclinic network (Rabinovich et al., 2000; Afraimovich et al., 2004b; Rabinovich et al., 2008). WLC was proposed as a general information processing principle for dynamical networks and is characterized by dynamic switching between network states, where the switching behavior is based on external input (Afraimovich et al., 2004b) (see Table 1). Importantly, the traveled trajectory identifies the received input, while any single state of the trajectory generally does not, see for example Neves and Timme (2012). In phase space representation, WLC can be achieved by open or closed sequences of heteroclinically concatenated saddle points. Such sequences are termed stable heteroclinic sequences (SHS) if the heteroclinic connections are dissipative, i.e., when a trajectory starting in a neighborhood close to the sequence remains close (Afraimovich et al., 2004a). While perturbations and external forcing can destroy stable heteroclinic sequences, it can be shown that even under such adverse circumstances, in many neurobiologically relevant situations the general sequential behavior of the system is preserved (Rabinovich et al., 2006). Such behavior is described by the concept of Stable Heteroclinic Channels (SHC) (see Figure 3 and Table 1) (Rabinovich et al., 2006). A simple implementation of SHCs is based on the generalized Lotka-Volterra equations (Bick and Rabinovich, 2010; Rabinovich et al., 2001), which are a type of recurrent neural network implicitly implementing the WLC concept. The temporal precision of a system that evolves along an SHC is defined by the noise level as well as the eigenvalues of the invariant directions of the saddle points. Therefore, sequences along heteroclinic trajectories are reproducible although the exact timing of the sequence elements may be subject to fluctuation.

In a similar approach, recent theoretical work on the behavior of RNNs has introduced the concept of excitable network attractors, which are characterized by stable states of a system connected by excitable connections (Ceni et al., 2019). The conceptual idea of orbits between fixed points may further be implemented in different ways. For instance, transient activation of neuronal clusters can be achieved by autonomously driven destabilization of stable fixed points (Gros, 2007, 2009).

### 2.3. Hierarchical Sequence Generators

**Figure 6:**
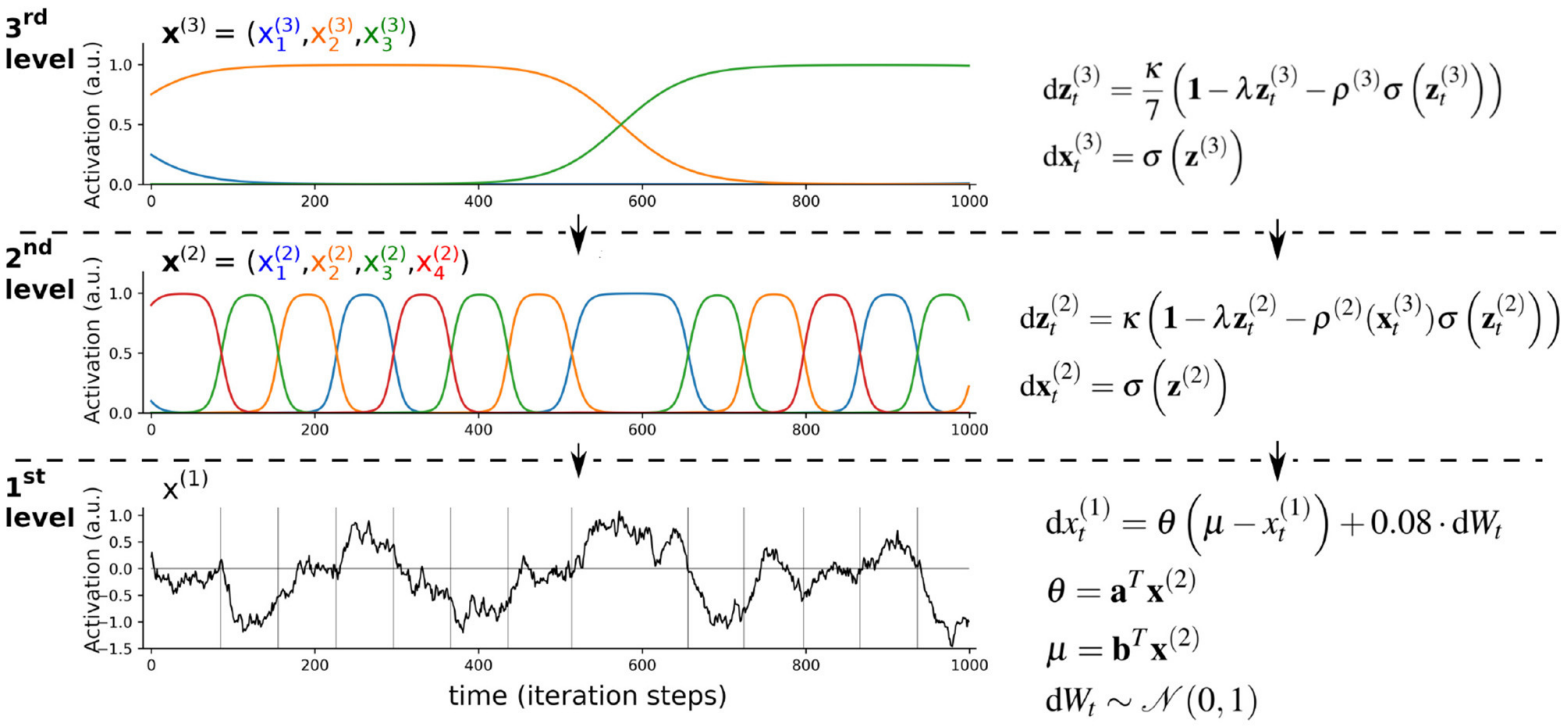
Illustration of hierarchical continuodiscrete dynamics based on limit cycles. Slowly changing dynamics at the 3rd level parametrize the sequence of states of the faster changing 2nd-level dynamics *z*^(2)^. As the dynamics of variables ${\vec{x}}^{\left(2\right)}$ and ${\vec{x}}^{\left(3\right)}$ change between the states “on” and “off,” their behavior constitutes continuodiscrete WLC dynamics. At around iteration step 600, the green unit at the 3rd level (element ${\vec{x}}_{3}^{\left(3\right)}$) becomes active, which changes the 2nd-level sequential dynamics from red→green→orange→blue→red to green→orange→red→blue→green. This is achieved by a change of the 2nd-level connectivity matrix *ρ*^(2)^ which depends on the 3rd-level variable ${\vec{x}}^{\left(3\right)}$. In this toy example, the 2nd-level dynamics model the evolution of the parameters of an Ornstein-Uhlenbeck process (black graph showing the evolution of variable *x*^(1)^). In the framework of hierarchical generative modeling, the 1st level would correspond to an agent's predictions of its sensory input, while the higher levels are the hidden states of the agent's generative model. This hierarchical parametrization of sequences is similar to the approach in Kiebel et al. (2009). The dot product between vectors ***b*** = (0.6, 0, −1, −0.3)^*T*^ and ${\vec{x}}^{\left(2\right)}$ determines the 1st-level attractor *μ*. The rate parameter Θ is parametrized by vector ***a*** = (1, 0.5, 1.2, 0.8)^*T*^ and its dot product with ${\vec{x}}^{\left(2\right)}$. σ(·) is the softmax function which is applied element-wise. **1** denotes a vector of ones. *κ* = 2, λ = 1/8. Gray vertical lines in the 1st level mark the time-points where states in the 2nd level change. This hierarchical parametrization of sequences is similar to the approach in Kiebel et al. (2009). Similar hierarchical autonomous models can be used as a generative model for Bayesian inference to achieve prediction and recognition of sequential data, as has for example been done in Yildiz and Kiebel (2011) and Yildiz et al. (2013).

As briefly introduced in section 1.2, growing evidence suggests that the brain is organized into a hierarchy of different time scales, which enables the representation of different temporal features in its sensory input (e.g., Murray et al., 2014; Hasson et al., 2008; Cocchi et al., 2016; Mattar et al., 2016; Gauthier et al., 2012). Here the idea is that lower levels represent dynamics at faster time scales, which are integrated at higher levels that represent slower time scales. For example, speech consists of phonemes (fast time scales), which are integrated into increasingly slower representations of syllables, words, sentences, and a conversation (Hasson et al., 2008; Ding et al., 2016; Boemio et al., 2005). The combination of this hierarchical aspect of brain function with the Bayesian brain hypothesis and the concept of neuronal sequences suggests that the brain implicitly uses hierarchical continuodiscrete dynamical systems as generative models. One illustrative example of a hierarchical continuodiscrete process is given in Figure 6. In this example, the dynamics of the 2nd and 3rd level of the hierarchy are modeled by limit cycles and govern the evolution of parameters of the sequence-generating mechanisms at the levels below. Such an approach for a generative model for prediction and recognition of sensory data has been used to model birdsong and human speech recognition (Yildiz and Kiebel, 2011; Yildiz et al., 2013; Kiebel et al., 2009) (see Figure 6). In Yildiz and Kiebel (2011), the 3rd level represented sequential neuronal activity in area HVC (proper name, see also Figure 1B), and the 2nd level modeled activity in the robust nucleus of the arcopallium (RA). Similarly, in Rivera et al. (2015) the authors employed a hierarchical generative model with a heteroclinic sequence for a sequence-generating mechanism to model odor recognition in the insect brain. In a slightly different approach to hierarchical continuodiscrete modeling, hierarchical SHCs, implementing winnerless competition, were used to demonstrate how chunking of information can emerge, similar to memory representation in the brain (Fonollosa et al., 2015). One computational study provided a proof of principle that complex behavior, like handwriting, can be decomposed into a hierarchical organization of stereotyped dynamical flows on manifolds of lower dimensions (Perdikis et al., 2011). These stereotyped dynamics can be regarded as the discrete points in a continuodiscrete sequence, which gave rise to complex and flexible behavior.

In the following section, we will briefly review how sequential methods have been used for problems in neuroscience and especially AI. Afterwards, we will review evidence for the organization of neuronal sequences into a hierarchy of time scales.

## 3. Recognition of Sequences

Although neuronal sequence models, such as the ones introduced in the preceding sections have been used to explain experimentally observed neuronal activity, these models by themselves do not explain how predictions are formed about the future trajectory of a sequence. To take the example of song production and recognition in songbirds, a sequence-generating model of birdsong generation is not sufficient to model or explain how a listening bird recognizes a song (Yildiz and Kiebel, 2011). Given a generative model, recognition of a song corresponds to statistical model inversion (Watzenig, 2007; Ulrych et al., 2001). A simple example of such a scheme is provided in Bitzer and Kiebel (2012), where RNNs are used as a generative model such that model inversion provides for an online recognition model. As shown in Friston et al. (2011), one can also place such a generative model into the active inference framework to derive a model that not only recognizes sequential movements from visual input but also generates continuodiscrete movement patterns. Generative models are not only interesting from a cognitive neuroscience perspective but also point at a shared interest with the field of artificial intelligence and specifically machine learning, to find a mechanistic understanding of how spatiotemporally structured sensory input can be recognized by an artificial or a biological agent. In the following, we will discuss how both fields seem to converge on the conceptual idea that generative models should be spatiotemporally structured and hierarchical.

### 3.1. Sequence Recognition in Machine Learning

The most widely-used models for discrete sequence generation are hidden Markov models (HMM) and their time-dependent generalisation, hidden semi-Markov models (HSMM) (Yu, 2015). In particular, HMMs and HSMMs are standard tools in a wide range of applications concerned with e.g., speech recognition (Liu et al., 2018; Zen et al., 2004; Deng et al., 2006) and activity recognition (Duong et al., 2005). Furthermore, they have often been used for the analysis of neuronal activity (Tokdar et al., 2010) and human behavior in general (Eldar et al., 2011). Similar to HSMMs, artificial RNNs are used in machine learning for classifying and predicting time series data. When training a generic RNN for prediction and classification of time series data, one faces various challenges, most notably incorporating information about long-term dependencies in the data. To address these dependencies, specific RNN architectures have been proposed, such as *long-short term memory* (LSTM) networks (Gers et al., 1999) and *gate recurrent units* (GRU) (Chung et al., 2014). In a common LSTM network, additionally to the output variable, the network computes an internal memory variable. This endows the network with high flexibility. LSTM networks belong to the most successful and most widely applied RNN architectures, with applications in virtually every field involving time-series data, or any data structure with long-range dependencies (Yu et al., 2019; LeCun et al., 2015). Another RNN approach is *reservoir computing* (RC), which started with the development of echo-state networks and liquid state machines in the early 2000s (Lukoševičius et al., 2012; Jaeger, 2001; Maass et al., 2002). In RC, sequential input is fed to one or more input neurons. Those neurons are connected with a *reservoir* of randomly connected neurons, which in turn are connected to one or more output neurons. Connections in the reservoir are pseudo-randomized to elicit dynamics at the edge of chaos (Yildiz et al., 2012), leading to a spatiotemporal network response in the form of reverberations over multiple time scales. RC networks have successfully been applied in almost every field of machine learning and data science, such as speech recognition, handwriting recognition, robot motor control, and financial forecasting (Lukoševičius et al., 2012; Tanaka et al., 2019).

While there is a lot of research on neurobiologically plausible learning paradigms for RNNs (Sussillo and Abbott, 2009; Miconi, 2017; Taherkhani et al., 2020), one possible approach for understanding the role of neuronal sequences is to use neurobiologically more plausible sequence generation models, which can act as generative models of the causal dynamic relationships in the environment. A natural application would be the development of recognition models based on Bayesian inference (Bitzer and Kiebel, 2012), and more specifically in terms of variational inference (Friston et al., 2006; Daunizeau et al., 2009).

### 3.2. Biological and Artificial Inferential Hierarchies

In neuroscience and the cognitive sciences, the brain is often viewed as a hierarchical system, where a functional hierarchy can be mapped to the structural hierarchy of the cortex (Badre, 2008; Koechlin et al., 2003; Kiebel et al., 2008). The best example of such a hierarchical organization is the visual system, for which the existence of both a functional and an equivalent structural hierarchy is established (Felleman and Van Essen, 1991). Cells in lower levels of the hierarchy encode simple features and have smaller receptive fields than cells further up the hierarchy, which posses larger receptive fields and encode more complex patterns by integrating information from lower levels (Hubel and Wiesel, 1959; Zeki and Shipp, 1988; Giese and Poggio, 2003). This functional hierarchy is mediated by an asymmetry of recurrent connectivity in the visual stream, where forward connections to higher layers are commonly found to have fast, excitatory effects on the post-synaptic neurons, while feedback connections act in a slower, modulatory manner (Zeki and Shipp, 1988; Sherman and Guillery, 1998). Moreover, neuroimaging studies have shown that the brain is generally organized into a modular hierarchical structure (Bassett et al., 2010; Meunier et al., 2009, 2010). This is substantiated by other network-theoretical characteristics of the brain, like its scale-free property (Eguiluz et al., 2005), which is a natural consequence of modular hierarchy (Ravasz and Barabási, 2003). Hierarchies also play an important role in cognitive neuroscience as most if not all types of behavior, as well as cognitive processes, can be described in a hierarchical fashion. For example, making a cup of tea can be considered a high-order goal in a hierarchy with subgoals that are less abstract and temporally less extended. In the example of making a cup of tea, these subgoals can be: (i) putting a teabag into a pot, (ii) pouring hot water into the pot, and (iii) pouring tea into a cup (example adopted from Botvinick, 2007).

#### 3.2.1. A Hierarchy of Time Scales

Importantly, all theories of cortical hierarchies of function share the common assumption that primary sensory regions encode rather quickly changing dynamics representing the fast features of sensory input, and that those regions are at the bottom of the hierarchy, while temporally more extended or more abstract representations are located in higher order cortices. This principle has been conceptualized as a “hierarchy of time scales” (Kiebel et al., 2008; Hasson et al., 2008; Koechlin et al., 2003; Badre, 2008; Kaplan et al., 2020). In this view, levels further up the hierarchy code for more general characteristics of the environment and inner cognitive processes, which generally change slowly (Hasson et al., 2008; Koechlin et al., 2003; Badre, 2008). For example, although the visual hierarchy is typically understood as a spatial hierarchy, experimental evidence is emerging that it is also a hierarchy of time scales (Cocchi et al., 2016; Gauthier et al., 2012; Mattar et al., 2016). Importantly, the information exchange in such a hierarchy is bidirectional. While top-down information can be regarded as the actions of a generative model trying to predict the sensory input (Dayan et al., 1995; Friston, 2005), recognition is achieved by bottom-up information that provides higher levels in the hierarchy with information about the sensory input, see also Yildiz and Kiebel (2011) and Yildiz et al. (2013) for illustrations of this concept. A related finding is an experimentally observed hierarchy of time scales with respect to the time lag of the autocorrelation of neuronal measurements (e.g., Murray et al., 2014). Here, it was found that the decay of autocorrelation was fastest for sensory areas (<100 ms) but longest for prefrontal areas like ACC (>300 ms).

The importance of cognition based on spatiotemporal structure at multiple time scales is also illustrated by various computational modeling studies. In one study, robots were endowed with a neural network whose parameters were let free to evolve over time to optimize performance during a navigation task (Nolfi, 2002). After some time, the robots had evolved neural assemblies with representations at clearly distinct time scales: one assembly had assumed a quickly changing, short time scale associated with immediate sensory input while another assembly had adopted a long time scale, associated with an integration of information over an extended period of time, which was necessary for succeeding at the task. Another modeling study showed that robots with neuronal populations of strongly differing time-constants performed their tasks significantly better than when endowed only with units of approximately identical time-constants (Yamashita and Tani, 2008). In Botvinick (2007) it was shown that, after learning, a neural network with a structural hierarchy similar to the one proposed for the frontal cortex had organized in such a way that high-level units coded for temporal context while low-level units encoded fast responses similar to the role assigned to sensory and motor regions in theories of hierarchical cortical processing (Kiebel et al., 2008; Alexander and Brown, 2018; Rao and Ballard, 1999; Botvinick, 2008; Badre, 2008; Koechlin et al., 2003; Fuster, 2004).

The principle of representing spatiotemporal dynamics at multiple time scales has also been used to model birdsong generation and inference in songbirds by combining a hierarchically structured RNN with a model of songbirds' vocal tract dynamics (Yildiz and Kiebel, 2011). The system consisted of three levels, each of which was governed by the sequential dynamics of an RNN following a limit cycle. The sequential dynamics were influenced both by top-down predictions, and bottom-up prediction errors. In another study, the same concept was applied to the recognition of human speech (Yildiz et al., 2013). The resulting inference scheme was able to recognize spoken words, even under adversarial circumstances like accelerated speech, since it inferred and adapted parameters in an online fashion during the recognition process. The same principle can also be translated to very different types of input, see Rivera et al. (2015) for an example of insect olfaction.

#### 3.2.2. A Hierarchy of Time Scales: Neuroimaging Evidence

**Figure 7:**
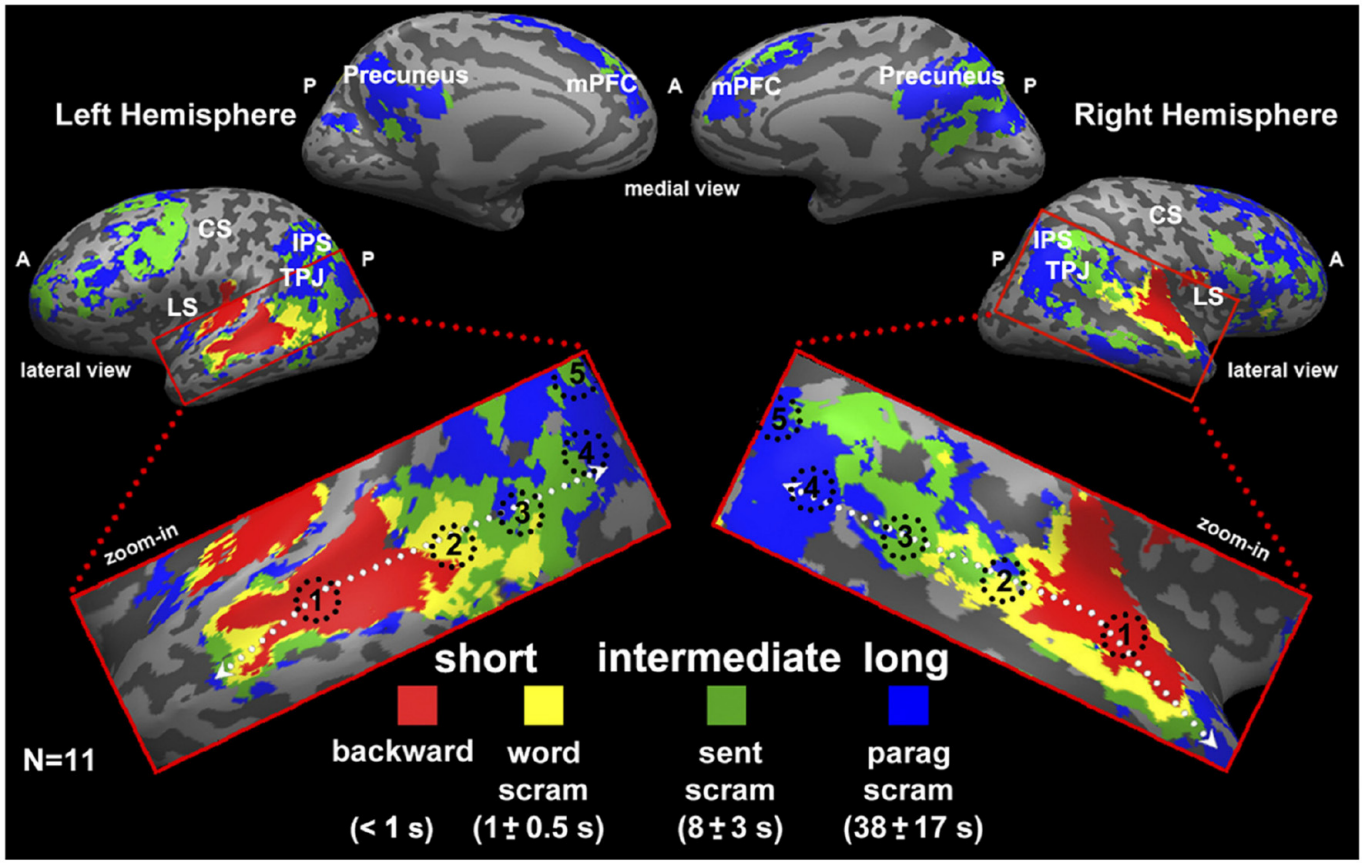
Study by Lerner et al. (2011) as an example for representations in a hierarchy of time scales. Here, the authors used fMRI and a between-subject correlational analysis to categorize brain voxels according to four levels of representation. These four levels were fast dynamics of auditory input (red), words (yellow), sentences (green), and paragraphs (blue). Results are displayed on a so-called inflated cortical surface. Figure reprinted from Lerner et al. (2011).

Experimental evidence for the hypothesis of a hierarchy of time scales has been reported in several neuroimaging studies (Koechlin et al., 2003; Hasson et al., 2008; Lerner et al., 2011; Gauthier et al., 2012; Cocchi et al., 2016; Mattar et al., 2016; Baldassano et al., 2017; Gao et al., 2020), two of which we are going to briefly discuss in the following. One functional magnetic resonance imaging (fMRI) study investigated the temporal receptive windows (TRW) of several brain regions in the human brain (Hasson et al., 2008). The TRW of an area is the time-interval over which the region “integrates” incoming information, in order to extract meaning over a specific temporal scale. It was found that regions, such as the primary visual cortex exhibited rather short TRW, while high order regions exhibited intermediate to long TRW (Hasson et al., 2008). Similarly, in Lerner et al. (2011) the same principle was tested with temporally structured auditory input, i.e., speech. Using fMRI, the authors found evidence for a hierarchy of time scales in specific brain areas. The different time scales represented fast auditory input, words, sentences and paragraphs (see Figure 7).

#### 3.2.3. A Hierarchy of Time Scales: Machine Learning

Not surprisingly, the importance of hierarchies of time scales is well-established within the machine learning community (El Hihi and Bengio, 1996; Malhotra et al., 2015). Current state-of-the-art RNN architectures used for prediction and classification of complex time series data are based on recurrent network units organized as temporal hierarchies. Notable examples are the clockwork RNN (Koutnik et al., 2014), gated feedback RNN (Chung et al., 2015), hierarchical multi-scale RNN (Chung et al., 2016), fast-slow RNN (Mujika et al., 2017), and higher order RNNs (HORNNs) (Soltani and Jiang, 2016). These modern RNN architectures have found various applications in motion classification (Neverova et al., 2016; Yan et al., 2018), speech synthesis (Wu and King, 2016; Achanta and Gangashetty, 2017; Zhang and Woodland, 2018), recognition (Chan et al., 2016), and other related areas (Liu et al., 2015; Krause et al., 2017; Kurata et al., 2017). These applications of hierarchical RNN architectures further confirm the relevance of hierarchically organized sequence generators for capturing complex dynamics in our everyday environments.

## 4. Conclusion

Here, we have reviewed the evidence that our brain senses its environment as sequential sensory input, and consequently, uses neuronal sequences for predicting future sensory input. Although the general idea that the brain is a prediction device has by now become a mainstream guiding principle in cognitive neuroscience, it is much less clear how exactly the brain computes these predictions. We have reviewed results from different areas of the neurosciences that the brain may achieve this by using a hierarchy of time scales, specifically a hierarchy of sequential dynamics. If this were the case, the question would be whether already known neuroscience results in specific areas can be re-interpreted as evidence for the brain's operations in such a hierarchy of time scales. Such an interpretation is quite natural for neuroscience fields like auditory processing, where such a temporal hierarchy is most evident. But it is much less evident for other areas, like for example decision-making. To further test this suggested theory of brain function, researchers need to design experimental paradigms which are specifically geared toward testing what probabilistic inference mechanisms the brain uses to predict its input at different time scales, and select its own actions. Importantly, hierarchical computational modeling approaches as reviewed here could be used to further provide theoretical evidence of the underlying multi-scale inference mechanism and generate new predictions that can be tested experimentally.

What we found telling is that recent advances in machine learning converge on similar ideas of representing multi scale dynamics in sensory data, although with a different motivation and different aims. The simple reason for this convergence may be that much of the sensory data that is input to machine learning implementations is similar to the kind of sensory input experienced by humans, as for example in videos and speech data. Therefore, we believe that as computational modeling in the neurosciences as reviewed here will gain traction, there will be useful translations form the neurosciences to machine learning applications.

## Author Contributions

DM and SK contributed to the conception of the manuscript. SF wrote the manuscript, with contributions by DM and SK. All authors contributed to the article and approved the submitted version.

## Conflict of Interest

The authors declare that the research was conducted in the absence of any commercial or financial relationships that could be construed as a potential conflict of interest.
